# Effects of Coenzyme Q10 and Diamond Nanoparticles on Ischemia-Reperfusion-Induced Testicular Damages in Rats

**DOI:** 10.31661/gmj.v10i0.2029

**Published:** 2022-02-05

**Authors:** Masoumeh Masoumi, Mitra Salehi, Seyed Abdolhamid Angaji, Mehrdad Hashemi

**Affiliations:** ^1^Department of Genetics, Faculty of biosciences, Islamic Azad University, North Tehran Branch, Tehran, Iran; ^2^Department of Microbiology, Faculty of biosciences, Islamic Azad University, North Tehran Branch, Tehran, Iran; ^3^ Department of Cell and Molecular Biology, Faculty of Biosciences, Kharazmi University, Tehran, Iran; ^4^Department of Genetics, Faculty of Advanced Science and Technology, Islamic Azad University, Tehran, Iran; ^5^Farhikhtegan Medical Convergence Sciences Research Center, Farhikhtegan Hospital Tehran Medical Sciences, Islamic Azad University, Tehran, Iran

**Keywords:** Rats;, CoQ10;, Oxidative Stress;, Testicle, Diamond Nanoparticles, Ischemia-Reperfusion

## Abstract

**Background:**

Ischemia-reperfusion (I/R) induced by testicular torsion can damage the testicles. In the present study, we assessed the effects of coenzyme Q10 (CoQ10) and diamond nanoparticles on sperm parameters in I/R testes in rats.

**Materials and Methods:**

Forty-eight Wistar adult male rats were divided into eight groups: healthy control (Ch), diamond nanoparticle healthy control group (Ch+Dia), CoQ10 healthy control group (Ch+Q10), diamond nanoparticles+CoQ10 healthy control group (Ch+Q10+Dia), torsion/detorsion group (Ct), the Ct group that received diamond nanoparticles (Ct+Dia), the Ct group that received CoQ10 (Ct+Q10), and Ct group that received diamond nanoparticles and CoQ10 (Ct+Q10+Dia). The rats were euthanized, and we collected the semen from the epididymal tissues to evaluate sperm viability, motility, concentration, and morphology parameters.

**Results:**

The I/R of the testicles significantly reduced sperm concentration, motility, viability, and altered sperm morphology in the rats. However, the administration of CoQ10 significantly improved sperm parameters in the rats with testicular I/R. Diamond nanoparticles decreased the sperm parameters; however, simultaneous administration of diamond nanoparticles and CoQ10 led to improved sperm parameters.

**Conclusion:**

CoQ10 potentially appeared to have protective effects against the long-term side-effects of I/R in testes in rats. Co-administration of diamond nanoparticles with CoQ10 significantly improved sperm parameters and greatly reduced the negative effects of diamond nanoparticles alone. Therefore, green synthesis of nanoparticles with the use of antioxidants such as CoQ10 is recommended.

## Introduction

Sperm production occurs inside the seminiferous tubules, after which sperm are transferred to the epididymides for storage. Male Infertility is dependent on various parameters such as the number, motility, viability, and morphology of sperm, which are influenced by several factors [1]. There are several reasons why the blood may not reach the testicular tissue, which may be due to the lack of oxygen to the tissue that causes hypoxia and the accumulation of large amounts of material [1]. Injury to the testicles due to trauma or torsion requires emergency surgical intervention [2]. Impaired testicular blood flow, following primary trauma or due to surgical procedures, can result in testicular cell death due to depletion of stored energy in the cells and the accumulation of toxic metabolites [3]. On the other hand, postoperative bleeding and oxidative stress exacerbate ischemic damage [4]. Oxidative damage has a detrimental effect on DNA, and it impairs protein function and increases membrane lipid peroxidation [5]. Mammalian sperm are rich in unsaturated fatty acids; therefore, they are very sensitive to reactive oxygen species (ROS), which results in reduced male fertility [5]. Cell death followed by ischemia-reperfusion (I/R) is closely associated with free radical production and the resultant lipid peroxidation [5]. Although there is no available definitive treatment for I/R injury, recently proposed interventions include blockage of the pathways of free radicals, anti-inflammatory medications, angiotensin-converting enzyme inhibitors, adenosine, morphine, and statins [6]. tudies show that antioxidant compounds and free radical scavengers have protective effects against the damage caused by the I/R process [7]. Coenzyme Q10 (CoQ10) is a cofactor that produces adenosine triphosphate (ATP) during oxidative phosphorylation in mitochondria [7]. Due to the role of this coenzyme in energy production, it has clinical applications in highly active metabolic tissues, such as the heart [7]. It has been suggested that the antioxidant ubiquinol (a reduced form of CoQ10) can be recycled to produce other antioxidants in the body [7]. This compound plays a role in other cellular processes such as gene expression, membrane stability, and cellular signaling [7]. CoQ10 is naturally present in mitochondria cells and is thought to protect cells from damage and abnormal growth [8]. CoQ10 is an antioxidant with energy-boosting and anti-inflammatory properties that can protect cells against apoptosis [9]. There is the widespread use of nanoparticles in various fields. Because of their ability to pass through cell membranes, it is necessary to investigate the effects of nanoparticles on testicular tissue [10]. The results of studies show that low doses of nanoparticles have antioxidant properties and reduce apoptosis, whereas high doses are toxic and increase apoptosis [10]. In addition, the concomitant use of nanoparticles with antioxidants improves their performance [11]. Diamond nanoparticles can be used as a stable drug delivery system for antibody therapy [12] or implant cover [13]. Nanodiamond-based drugs have been used for local drug delivery and in treatments for advanced tumors [14]. Due to the significant effect of free radicals in causing I/R lesions, the combination of CoQ10 with diamond nanoparticles appears to be effective in reducing both inflammatory precursors and I/R damage to the testicles. Therefore, it is necessary to find solutions that improve the treatment process. Hence, the present study aimed to investigate the effect of CoQ10, as an antioxidant, and diamond nanoparticles on testicular function in rats with I/R damages.

## Materials and Methods

### Chemical and Agents

We purchased diamond nanoparticles (purity: >97%, particle size: 4 nm, density 3.18) from Sigma Aldrich (Germany). The injectable drug Q10 (CoQ10 red) was obtained from the Antiaging Institute (California, USA). We determined the dose of diamond nanoparticles based on the median lethal dose (LD50), which is the concentration that caused the death of half of the rats. Accordingly, 0.005, 0.01, 0.02, 0.03, 0.04, and 0.05 mg/kg body weight concentrations were given to the rats. Based on the results, the LD50 was determined to be 0.02 mg/kg body weight. Therefore, we used this concentration for the subsequent experiments.

### Animals

We obtained 48 adult male rats from Pasteur Institute (Tehran, Iran). The animals were kept under standard conditions on a 12-hour light/dark schedule, a temperature of 25±2°C, and relative humidity of 50±10%. All animals were fed the same proportions of corn, wheat, barley, and pellets under the same nutritional conditions and had unlimited access to water.

### Induction of Torsion

The rats were anesthetized with ketamine (50 mg/kg) and xylazine (5 mg/kg) according to a protocol approved by the Committee for the Conservation and Use of Animals [15]. Torsion-detorsion was performed by rotating the testicles 720° counterclockwise for 90 minutes. In the experimental and control groups that underwent torsion, detorsion continued for ten days when treatments were applied [2].

### Design and Treatment

After torsion-detorsion of the testicles, the presence of severe oligoasthenoteratozoospermia was confirmed by pathological analysis. Subsequently, we randomly divided the rats into eight groups: healthy control (Ch), Ch group that received diamond nanoparticles (Ch+Dia; 0.02 mg/kg body weight [BW]), Ch group that received CoQ10 (Ch+Q10; 0.02 mg/kg BW), Ch+Q10 group that received diamond nanoparticles (Ch+Q10+Dia; 0.02 mg/kg BW), torsion/detorsion (Ct), Ct group that received diamond nanoparticles (Ct+Dia; 0.02 mg/kg BW), Ct group that received CoQ10 (Ct+Q10; 0.02 mg/kg BW), Ct+Q10 group that received diamond nanoparticles (Ct+Q10+Dia; 0.02 mg/kg BW). All of the animals were euthanized by an overdose of anesthesia after the completion of the treatments. Their testicular tissues were removed for the tissue assessments, and we collected the semen samples from the epididymides for analysis of sperm and cellular parameters.

### Sperm Analysis

We evaluated four attributes for sperm analysis include morphology, viability, concentration, and motility. Sperm (10 μL) were transferred to a hemocytometer, and we counted the diluted sperm under an optical microscope (Olympus, Tokyo, Japan) at 40x magnification [16]. We evaluated sperm motility by microscopic observation of ten fields based on recommendations by the World Health Organization. For this purpose, 200 sperm cells were counted to determine the percentage of motile sperm [16]. Eosin-Nigrosin (Farzaneh Arman Co, Iran) staining was used to evaluate sperm viability. Sperm morphology was assessed using a standard Papanicolaou stain protocol [16], and the numbers of normal and abnormal sperm were determined under a microscope at 100x magnification. We evaluated 200 sperm for the presence of abnormal morphology, which was defined as sperm with two heads, large head, small head, round head, no acrosome, no head, long or short tail, no tail or twisted tail, and cytoplasmic diameter.

### Ethical Consideration

This project has been done with a code IR.IAU.TNB.REC.1399.024 in accordance with ethical principles and national norms and standards for conducting medical research in Islamic Azad University- North Tehran Branch.

### Statistical Analysis

One-way analysis of variance (ANOVA) was used to identify significant differences in the studied characteristics among the rat groups. SPSS software version 22 (IBM SPSS Statistics for Windows, Version 22.0. Armonk, NY: IBM Corp) was used for data analysis. P<0.05 was considered to be statistically significant.

## Results

**Figure 1:**
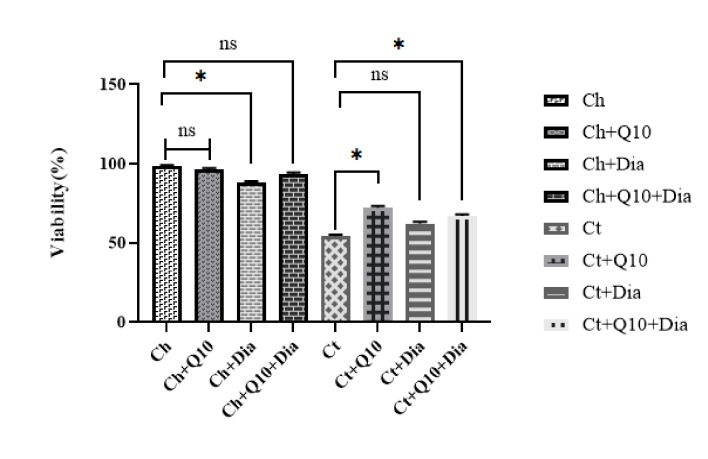
The effect of CoQ10 (Q10) and diamond nanoparticles (Dia) on improvement in the percentage of viable sperm in the different rat groups. * P<0.05. **Ch:** Healthy control; **Ch+Q10:** Healthy control receiving CoQ10;** Ch+Dia: **Healthy control receiving Diamond;** Ch+Q10+Dia: **Healthy control receiving CoQ10+Diamond;** Ct:** Torsion/detorsion;** Ct+Q10:** Torsion/detorsion receiving CoQ10;** Ct+Dia:** Torsion/detorsion receiving Diamond;** Ct+Q10+Dia: **Torsion/detorsion receiving CoQ10+Diamond.

**Figure 2:**
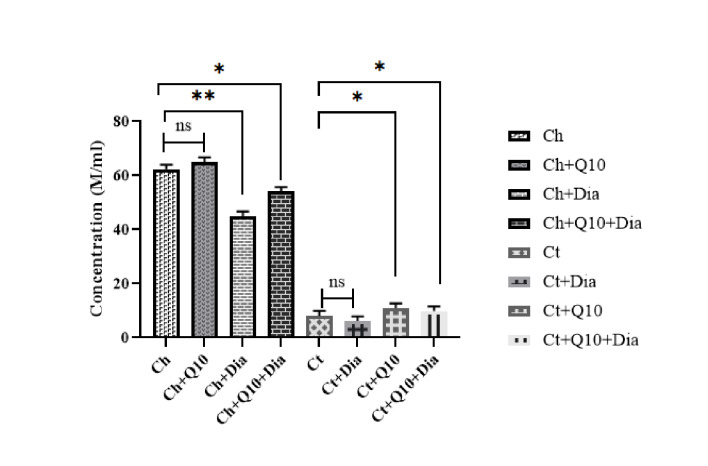
The effect of CoQ10 (Q10) and diamond nanoparticles (Dia) on improved sperm concentrations in the different rat groups. * and ** indicate significant differences at the probability level of P<0.05 and P<0.01, respectively. **Ch: **Healthy control; **Ch+Q10: **Healthy control receiving CoQ10; **Ch+Dia: **Healthy control receiving Diamond; **Ch+Q10+Dia: **Healthy control receiving CoQ10+Diamond; **Ct: **Torsion/detorsion; **Ct+Q10: **Torsion/detorsion receiving CoQ10; **Ct+Dia: **Torsion/detorsion receiving Diamond; **Ct+Q10+Dia: **Torsion/ detorsion receiving CoQ10+Diamond.

**Figure 3:**
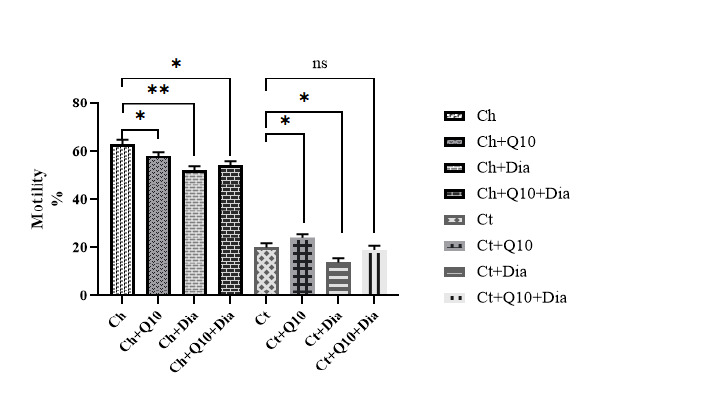
The effect of CoQ10 (Q10) and diamond nanoparticles (Dia) on improving sperm motility in different rat groups. * and ** indicate significant differences at the probability level of P<0.05 and P<0.01, respectively. **Ch: **Healthy control; **Ch+Q10: **Healthy control receiving CoQ10; **Ch+Dia: **Healthy control receiving Diamond; **Ch+Q10+Dia: **Healthy control receiving CoQ10+Diamond; **Ct: **Torsion/ detorsion; **Ct+Q10: **Torsion/detorsion receiving CoQ10; **Ct+Dia: **Torsion/detorsion receiving Diamond; **Ct+Q10+Dia: **Torsion/detorsion receiving CoQ10+Diamond.

**Figure 4:**
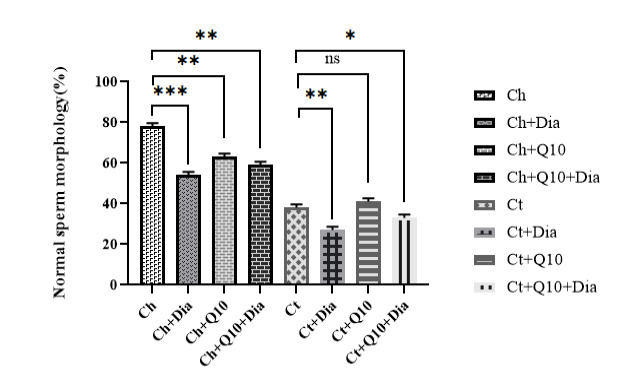
The effect of CoQ10 (Q10) and diamond nanoparticles (Dia) on sperm morphology in the different rat groups. *, **, and *** indicate significant differences at a probability level of P<0.05, P<0.01, and P<0.001, respectively. **Ch: **Healthy control; **Ch+Q10: **Healthy control receiving CoQ10; **Ch+Dia: **Healthy control receiving Diamond; **Ch+Q10+Dia: **Healthy control receiving CoQ10+Diamond; **Ct: **Torsion/detorsion; **Ct+Q10: **Torsion/detorsion receiving CoQ10; **Ct+Dia: **Torsion/detorsion receiving Diamond; **Ct+Q10+Dia: **Torsion/ detorsion receiving CoQ10+Diamond.

**Figure 5:**
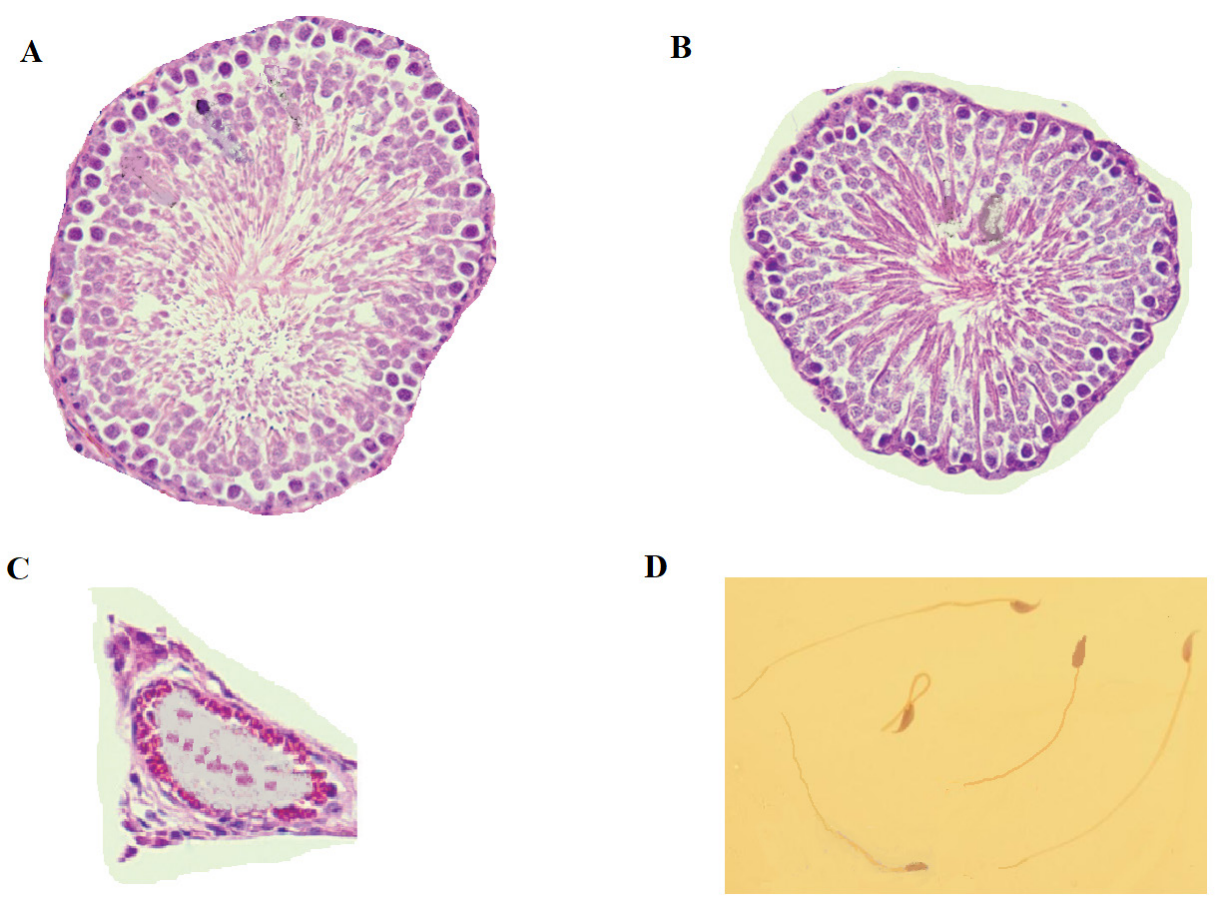
Seminiferous tubule morphology.** A: **Healthy control rats;** B: **Testicular ischemia rats that were receiving Q10; **C:** The control testicular ischemia rats;** D: **Spermatozoa stained with standard Papanicolaou stain (original magnification: 400 x, H&E stain x 200).

Sperm Viability Percentage There were significant differences in sperm viability in the different groups according to one-way ANOVA (P<0.001). CoQ10 had a significant effect on increasing the percentage of viable sperm. In terms of sperm viability, untreated rats with ischemic testicles had the lowest percentage of viable sperm. This percentage increased significantly in the ischemic rats that received CoQ10. We examined the effect of diamond nanoparticles on testicular ischemia and noted a decreased effect of diamond nanoparticles on the rate of viable sperm. Sperm viability was lower in rats with ischemic testicles that received the diamond nanoparticles compared with the group with ischemic testicles that received CoQ10. Concomitant administration of CoQ10 and diamond nanoparticles increased sperm viability (Figure-1). Sperm Concentration ANOVA analysis indicated significant differences between the different groups in terms of sperm concentration (P<0.001). Rats with ischemic testicular had very low sperm concentrations, which indicated a sharp decrease in sperm concentration under ischemic conditions. However, rats with testicular ischemia were given CoQ10 and CoQ10 with diamond nanoparticles; there was a relative improvement in sperm concentration. The highest sperm concentrations were observed in Ch and Ch+Q10 groups, whereas the lowest concentrations were observed in the group of rats with testicular ischemia (Ct) and those in the Ct+Dia group (Figure-2). These results indicated relative improvements in sperm concentration following treatment with CoQ10. Sperm Motility In terms of the percentage of sperm motility, one-way ANOVA results showed significant differences among the different groups (P<0.001). The highest percentage of sperm mobility was observed in the Ch group and Ch+Q10 groups. The lowest sperm motility was observed in the Ct+Dia group. However, we observed increased sperm motility in rats with ischemic testicles when diamond nanoparticles were co-administered with CoQ10. In the group of rats with testicular ischemia, the highest sperm motility was observed in the Ct+Q10 group (Figure-3). Sperm Morphology The Ch+Q10 group had the highest normal sperm morphology. However, there was a substantial reduction in normal sperm morphology in the Ch+Dia group. Rats in the Ch+Q10+Dia had higher normal sperm morphology. In the rats with ischemic testicles, the lowest normal sperm morphology was observed in the Ct+Dia group, and the highest percentages of normal sperm morphology were observed in the rats of Ct+Q10 and Ct+Q10+Dia groups (Figure-4). The alteration in seminiferous tubule morphology was also evaluated, and the results indicated that its structure was abnormal in testicular ischemia rats compared to controls (Figure-5).

## Discussion

Blockage of the blood supply to an organ can quickly lead to cell damage and death [17]. Although reperfusion can save ischemic tissues, it also causes additional injury [17]. Several factors, such as increased production of ROS, free radicals, lipid peroxidation, cell death due to apoptosis or necrosis, inflammatory cytokines, and damage to small blood vessels, may play a role in post-I/R injuries [17]. The results of studies show that reperfusion of ischemic tissues leads to ROS formation [18]. In addition to ROS that causes membrane damage by lipid peroxidation, activation of phospholipase A2 stimulates leukocyte activity and induces cytotoxicity, increasing leukocyte adhesion to the endothelium after I/R [19]. Testicular ischemia also causes sperm cell death mainly due to the lack of oxygen for metabolic activities, depletion of stored cellular energy, and accumulation of toxic metabolites [4]. In the reperfusion phase, increased production of ROS and reactive nitrogen species significantly exacerbate ischemic cell damage in testicular tissue [4]. The current study showed that CoQ10 could improve sperm parameters in ischemic testicles. The reduction in sperm parameters following I/R has been attributed to the peroxidation of membrane lipids and oxidative damage. Our results indicated improved sperm parameters in the rats that received CoQ10, which was consistent with the results from other studies [20,21]. Administration of CoQ10 to pigs with cardiac I/R injury resulted in protective effects and a decreased myocardial infarction area compared with the control group [22]. CoQ10 has been shown to reduce the production of malondialdehyde during the reperfusion period, which indicated a reduction in membrane lipid peroxidation [21]. Therefore, the improvement of sperm parameters in the current study after administration of CoQ10 could be attributed to the reduction in membrane lipid peroxide. However, the effect of CoQ10 on reducing lipid peroxidation is time-dependent; although its long-term use has a protective effect, short-term use has not been shown to reduce lipid peroxidation in ischemic rats [21]. Therefore, long-term treatment with CoQ10 is essential to prevent the formation of lipid peroxidation products such as malondialdehyde. In the present study, we have observed that CoQ10 administration improved sperm parameters in rats with I/R testicles. This compound is an antioxidant and also inhibits lipid peroxidation [23]. The current study results revealed that treatment with CoQ10 prevents further reduction of semen parameters from I/R. Therefore, it concluded that this compound prevents oxidative damage in the testicles. We observed reduced semen parameters, altered sperm morphology, and reduced numbers and viability of sperms in rats with I/R testicles after administration of diamond nanoparticles. One study found that treatment of Wistar rats with silver nanoparticles resulted in a significant reduction in sperm counts [24]. The results of another study showed changes in sperm count and morphology [25], which was in line with the current study's findings. In another study, there was a sharp decrease in sperm mobility and viability and an increase in abnormal sperm production after treatment with zinc-oxide nanoparticles; these nanoparticles' toxic effect was dose-dependent [26]. Gold nanoparticles also caused a sharp decrease in sperm motility and severe changes in sperm morphology [27]. The results of the present study support the above-mentioned studies. The mechanism of nanoparticle damage to the testes has been attributed to oxidative damage, which ultimately reduces the thickness of the seminiferous epithelium in the testicles [28]. Therefore, the reduction in sperm parameters and changes in sperm morphology in the current study could be attributed to oxidative stress caused by diamond nanoparticles. However, in the present study, we observed that concomitant use of CoQ10 with diamond nanoparticles reduced the toxic effects of these nanoparticles on sperm parameters, which we attributed to the antioxidant properties of CoQ10. Cell death due to the presence of nanoparticles occurs by autophagy [29], and the results of the current research have shown that simultaneous use of CoQ10 with diamond nanoparticles improved the sperm parameters. Therefore, the antioxidant properties of CoQ10 enabled it to improve sperm parameters by reducing sperm cell autophagy. However, additional research is needed in this area. In recent years, the green synthesis of nanoparticles by plants has attracted much attention and it is considered to an alternative to the chemical methods of nanoparticle synthesis [30]. Green synthesis of nanoparticles is cost-effective. Therefore, we recommend the concomitant use of green synthesized diamond nanoparticles and CoQ10 in patients with testicular ischemia.

## Conclusion

The significant antioxidant effects of concomitant use of CoQ10 with diamond nanoparticles could improve the adverse effects of I/R testicles and be an appropriate option to improve treatment strategies that reduce fertility disorders caused by torsion of reproductive organs. However, the clinical features of simultaneous use of CoQ10 and diamond nanoparticles as a treatment for testicular torsion require clinical trial verification.

## Acknowledgment

None.

## Conflict of Interest

In this study, there is no financial support and conflict of interest.
